# The perception of Italian pregnant women and new mothers about their psychological wellbeing, lifestyle, delivery, and neonatal management experience during the COVID-19 pandemic lockdown: a web-based survey

**DOI:** 10.1186/s12884-021-03904-4

**Published:** 2021-07-01

**Authors:** Viviana Stampini, Alice Monzani, Silvia Caristia, Gianluigi Ferrante, Martina Gerbino, Alberto De Pedrini, Roberta Amadori, Ivana Rabbone, Daniela Surico

**Affiliations:** 1 Obstetrics and Gynecology Unit, Department of Translational Medicine, University of Eastern Piedmont, Via Solaroli 17, 28100 Novara, Italy; 2 Division of Pediatric, Department of Health Sciences, University of Eastern Piedmont, Novara, Italy; 3 Obstetrics and Gynecology Unit, Maggiore della Carità Hospital, Novara, Italy; 4 Department of Translational Medicine, University of Eastern Piedmont, Novara, Italy; 5 CPO Piemonte, Turin, Italy

**Keywords:** COVID-19, Lockdown, Pregnancy, New mothers, Breastfeeding, Healthy eating, Physical exercise.

## Abstract

**Background:**

In response to the COVID-19 pandemic, drastic measures for social distancing have been introduced also in Italy, likely with a substantial impact in delicate conditions like pregnancy and puerperium. The study aimed to investigate the changes in lifestyle, access to health services, and mental wellbeing during the first Italian lockdown in a sample of Italian pregnant women and new mothers.

**Methods:**

We carried out a web-based survey to evaluate how pregnant women and new mothers were coping with the lockdown. We collected data about healthy habits (physical exercise and dietary habits), access to health services (care access, delivery and obstetric care, neonatal care, and breastfeeding), and mental wellbeing (psychological well-being and emotive support). Descriptive analysis was performed for both groups of participants, whereas a Poisson analysis was used to measure the association between some structural variables (age, education, socio-economic data, partner support, contact, free time, previous children, and pregnancy trimester) and anxiety or depression, difficulties in healthy eating and reduction in physical activity after lockdown started. Chi2 and Adjusted Prevalence Ratios were estimated only for pregnant women.

**Results:**

We included 739 respondents (response rate 85.8 %), 600 were pregnant (81.2 %), and 139 (18.8 %) had delivered during lockdown (new mothers). We found a high score for anxiety and depression in 62.8 % of pregnant women and 61.9 % of new mothers. During the lockdown, 61.8 % of pregnant women reduced their physical exercise, and 44.3 % reported eating in a healthier way. 94.0 % of new mothers reported to have breastfed their babies during the hospital stay. Regarding the perceived impact of restrictive measures on breastfeeding, no impact was reported by 56.1 % of new mothers, whereas a negative one by 36.7 %.

**Conclusions:**

The high prevalence of anxiety and depressive symptoms in pregnant women and new mothers should be a public health issue. Clinicians might also recommend and encourage “home” physical exercise. On the other hand, about half of the sample improved their approach towards healthy eating and a very high breastfeeding rate was reported soon after birth: these data are an interesting starting point to develop new strategies for public health.

**Supplementary Information:**

The online version contains supplementary material available at 10.1186/s12884-021-03904-4.

## Background

Since the severe acute respiratory syndrome SARS-CoV-2 started to spread across several countries, the World Health Organization declared that the outbreak was a public health emergency of international concern (http://www.euro.who.int/en/health-topics/health-emergencies/coronavirus-covid-19/news/news/2020/3/who-announces-covid-19-outbreak-a-pandemic). Based on the Chinese experience [1], starting from March 9th, 2020, drastic measures have been introduced also in Italy: citizens were banned from leaving their homes unless for urgent needs. As a consequence, a sudden and radical change in habits and lifestyles of the whole population, a minimization of socialization, and changes in both interpersonal relationships and organization of work occurred. Hospital activity was radically changed: many departments were closed to create COVID-19 dedicated hospital wards, the rest of the clinical activity was downsized, and contacts with patients were reduced to a minimum. Measures such as redefinition of care priorities and several restrictions would presumably lead to changes in the health of the population in the coming months or years.

There are still many unanswered questions regarding the effects of lockdown measures on pregnant women [2, 3]. Healthcare workers are facing an important challenge in terms of reshaping obstetric care to avoid unnecessary exposure to patients, without impairing the required attention. Although obstetric units have not diminished their working activity, there have been changes in territorial and hospital care. The pre-birth courses have been officially stopped; some screening tests have been performed much less frequently, due to reduced patient access or to difficulties in providing services. Family members and partner presence during important moments, such as ultrasound scans and hospitalization, has been reduced for safety reasons.

Most of the pregnant women and new mothers were forced into homebound isolation, often with other children to look after, without any domestic help [4]. On the other hand, some women may have welcomed the chance of working from home and, in some cases, they may have benefited from a greater presence of their partner.

It can be assumed that these changes influenced pregnancy, puerperium, and newborn management with consequences worthy of obstetrics consideration [3]. While published studies on the possible effects of COVID-19 disease in pregnant women and infants are increasing [5], there are only a few studies [6, 7, 8] dealing with the psychological effects of the pandemic on pregnant women and new mothers.

The purpose of this study was to describe the lifestyle, access to health services, and mental wellbeing of Italian pregnant women and new mothers during the first phase of lockdown (April – May, 2020). In addition, we aimed to assess the association between socio-demographic characteristics and living/housing conditions with (i) anxiety and depression, (ii) healthy eating habits, and (iii) physical exercise.

## Methods

### Study Design, setting and participants

A survey investigating lifestyle, access to health services, and mental wellbeing of Italian pregnant women and new mothers was conducted from April 9th, 2020 to May 3rd, 2020. It was a cross-sectional study based on an anonymous web survey to collect information through an online electronic questionnaire, accessible from smartphones, tablets, and personal computers.

### Survey methodology

To recruit respondents, a non-probabilistic snowball sampling approach was used, disseminating the weblink of the online survey through social media (Facebook and Instagram pages addressing new mothers and pregnant women) and newspaper sections on women issues. Individuals were directed via an electronic link to an online survey platform (Google Forms). Duplicate entries were avoided by asking people to provide their e-mail address at the end of the survey; duplicate entries having the same e-mail address were eliminated before the analysis and the first entry was kept. The survey was not displayed a second time once the responder had filled it in, but the link to pass it on to others was available. Responses to the survey were automatically captured into a database. All responses included a non-response option, such as “not applicable” or “rather not say” in order to avoid missing data.

### Ethical considerations

The survey was preceded by a fact sheet including information on what the research was about, the reason the research was being conducted, how the data would be used, how the privacy of the data would be maintained, and information in case the respondents changed their mind during the survey, along with contact details for further information. Afterward, consent to participate was obtained at the very beginning of the survey, as participants were required to check a box to indicate their consent before accessing the questionnaire. The participation in the survey was voluntary and anonymous. Approval was obtained from the local Ethical Committee (Comitato Etico Interaziendale Novara CE 71/20), which conformed to the principles embodied in the Declaration of Helsinki.

### Variables and data sources

Expected outcomes of the baseline analysis included different topics that could be influenced by the lockdown measures and were categorized in different analysis domains, related to pregnancy and puerperium.

For the pregnancy group, the analyzed domains were 1- psychological well-being and support; 2- physical exercise; 3- dietary habits; 4- access to care. Regarding puerperium, the analyzed domains were 5- psychological well-being and support; 6- delivery and obstetric care; 7- neonatal care and breastfeeding.

Due to the lack of validated questionnaires about this topic, the authors reviewed previous and current surveys [9] on the impact of the pandemic and included additional questions related to pregnancy and birth [10, 11, 12], developing an ad hoc questionnaire (Supplementary material). To investigate the psychological impact, we used the Patient Health Questionnaire for Depression and Anxiety (the PHQ-4)[13]. PHQ-4 is a four-item scale with a total score ranging from 0 to 12 that aims to identify the following categories of psychological distress: none (0–2), mild (3-5), moderate (6-8), and severe (9-12). The questionnaire was tested in a sample of voluntary pregnant women and new mothers with different characteristics (age, education, parity) who reviewed the questionnaire individually and provided verbal feedback, and it was also submitted to a panel of experts (psychologists, midwives, epidemiologists), for content validity and construct coherence. Completion time was about 15 min. The survey was performed according to the Checklist for Reporting Results of Internet E-Surveys (CHERRIES) [11].

The survey consisted of a common part including sociodemographic and psychological questions, addressing both pregnant women and new mothers, followed by a specific part dedicated either to pregnant women or women who gave birth during the lockdown, each divided into subsections of questions, addressing different analysis domains. The completion of only some items of the survey was mandatory for all who visited the site. All submitted questionnaires were complete for the mandatory items. Items in the survey were not randomized or alternated for different survey respondents. Certain questions only displayed based on responses to other questions.

### Statistical analysis

A descriptive analysis was carried out to report the main socio-demographic characteristics and the living and housing conditions of the respondents by frequency distribution, separately for pregnant women and new mothers. Among pregnant women, psychological aspects, living habits during lockdown, and access to care were described, and among new mothers, the experience related to childbirth during lockdown was reported.

Crude associations between socio-demographic characteristics, living and housing conditions, and (i) anxiety and depression, (ii) healthy eating habits, and (iii) physical exercise were assessed by the chi2 test, setting the level of significance at 0.05. Poisson regression models were used to assess the same associations, adjusted for the variables included in the analysis (age, education, economically satisfied, satisfied with their home, partner support, contact with other people, availability of free time, having other children at home, and trimester of pregnancy). Adjusted Prevalence Ratios (Adj PR) were calculated along with 95 % confidence intervals.

Response rates for both groups were very high (see below), so we excluded from the analysis missing data (following Complete Case Analysis) supposing a random mechanism generating missing data (Missing Completely At Random).

Statistical analysis was performed using Stata Statistical Software: Release 15. StataCorp LLC.

## Results

**Fig. 1:**
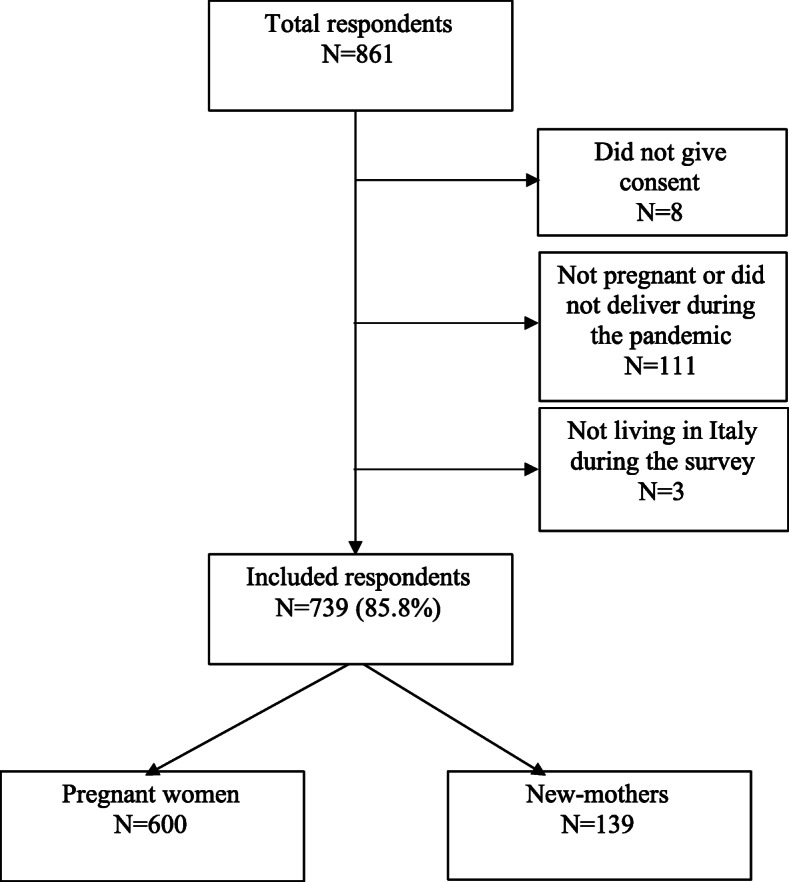
Respondents’ flowchart

The first page of the survey was visited by 861 women (Fig. 1). Eight (0.9 %) women did not give their consent to participate, 110 (12.8 %) were excluded because either not pregnant or not having delivered during lockdown, and 3 (0.5 %) because not living in Italy during the interview. Thus, we included 739 respondents from 18 different Italian regions, who completed the questionnaire (completion rate: 96 %). Overall, 600 respondents were pregnant (81.2 %) and 139 (18.8 %) had delivered in the lockdown period.

### Sociodemographic, living, housing, and psychological characteristics of pregnant women and new mothers

Data are reported in Table 1. The overall response rate for these items was > 99 %. Pregnant women were aged 18 to 48 years with a mean age of 33.1 ± 4.3, 61.6 % of the sample had at least a bachelor degree, 47.7 % of the sample reported being economically satisfied (highly sufficient to their needs). Most of them (81 %) were from Northern Italy. The PHQ-4 score ranged from moderate to severe (scores from 6 to 12) for 62.8 % of women. Finally, 83.2 % of women recognized their partner as the person who was supporting them during the lockdown.

**Table 1:** Socio-demographic characteristics, housing and living conditions in pregnant women and new mothers

	Parameter	Category	Pregnant women (***N***=600)		New mothers (***N***=139)	
			n	%	n	%
**Socio-demographic characteristics**	Age	From 18 to 34	379	63.2%	82	59.0%
		From 35 to 48	221	36.8%	57	41.0%
	Education	Less than Bachelor	230	38.4% ^a^	37	26.6%
		More than bachelor	369	61.6% ^a^	102	73.4%
	Area of residence	North	486	80.6%	125	89.9%
		Centre	65	10.8%	12	8.6%
		South	49	8.2%	2	1.4%
	City or village	City or suburbs of a city	282	47.2% ^a^	60	43.2%
		Village	316	52.8% ^a^	79	56.8%
	Economic resources	Not adeguate	312	52.0% ^a^	72	52.6% ^a^
		Very adeguate	285	47.7% ^a^	65	47.4% ^a^
	Working conditions	In-work	454	75.8% ^a^	108	78.3% ^a^
		Not in-work	145	24.2% ^a^	30	21.7% ^a^
**Housing**	House size	Less than 100 sm	387	64.5%	94	67.6%
		More than 100 sm	213	35.5%	45	32.4%
	Satisfied with the house	no	232	38.7%	54	38.8%
		yes	368	61.3%	85	61.1%
	Presence of a garden	Yes	253	42.2%	53	38.4%
		No	347	57.8%	85	61.6%
	Adequacy of electronic devices	Not adeguate	136	22.7%	41	29.5%
		Very adeguate	464	77.3%	98	70.5%
**Social-living**	Presence of partner	Always at home	311	51.8%	70	50.4%
		At home but still going to work	275	45.8%	65	46.8%
		Not co-living	14	2.3%	4	2.9%
	Other children at home	No	406	67.7%	99	71.2%
		Yes	194	32.3%	40	28.8%
	Someone else co-living	No	556	92.7%	123	88.5%
		Yes	44	7.3%	16	11.5%
	Contacts with other people	No	376	62.7%	71	51.4% ^a^
		Yes	224	37.3%	67	48.6% ^a^
	Adherence to the restrictions	From little to average	12	2.00%	1	0.7%
		High	588	98.0%	138	99.3%
**Psychological wellbeing and support**	Depression and anxiety score (PHQ-4 score) ^b^	Normal to mild	223	37.2%	53	38.1%
		Moderate to severe	378	62.8%	86	61.9%
	People supporting (more than one choice available)	Partner	499	83.2%	121	87.1%
		Mother	392	65.3%	92	36.7%
		Sister/brothers	231	38.5%	51	61.2%
		Friends	284	47.3%	60	43.2%
		Gynecologist	81	13.5%	7	5.0%
		Midwife	68	11.3%	27	19.4%
		Other women in pregnancy	112	18.7%	42	30.2%
		Websites	38	6.3%	2	1.4%

^a^ Presence of missing data for these variables. Percentages were calculated on total of respondents: PREGNANT WOMEN Education (*N*=599), City or village (*N*=598), Economic resources (*N*=597), Working conditions (*N*=599). NEW MOTHERS Economic resources (*N*=137), Working conditions (*N*=138); ^b^ PHQ-4 is a four items scale with a total score ranging from 0 to 12 and aims to identify the following categories of psychological distress: none (0-2), mild (3-5), moderate (6-8), and severe (9-12)

New mothers were aged 25 to 41 years with a mean of 33.6 ± 4.0, 73.4 % had at least a bachelor degree, and 47.4 % of the sample was economically satisfied. Similarly to pregnant women, for 61.9 % of new mothers the PHQ-4 score ranged from moderate to severe and the partner was the most cited supporting person (87.1 %). Almost the overall sample (89.9 %) was living in Northern Italy.

### Changes in the lifestyle of pregnant women during lockdown

Table 2 describes some aspects of pregnant women’s lifestyle and how the social distancing measures changed their habits (dietary habits, physical exercise, and access to care). The overall response rate for these items was > 96 %. Many women (79.0 %) declared that the greater presence of their partner positively influenced pregnancy. On the other hand, 71.7 % of them were afraid of delivering alone and 44.5 % declared they were living a stressful situation. Regarding physical exercise, the reported minutes of weekly physical activity were significantly decreased during lockdown (before: 142.2 min, 95 % CI 135.0–149.4, vs. after: 105.1 min, 95 % CI 96.7–113.4, with a mean reduction of 38.5 min ± 90.3 during the lockdown, *p* < 0.05). Specifically, 61.8 % of women reduced their physical exercise during the lockdown.

**Table 2:** Changes in lifestyle during the lockdown among pregnant women

	Parameter	Category	*N* = 600	%
Other psychological aspects	Influence of partner at home (*N* = 586) ^a^	Positive influence	444	79.0 %
		Negative influence	11	2.0 %
		No influence	107	19.0 %
	Influence of children at home (*N* = 194) ^a^	Positive influence	51	26.7 %
		Negative influence	70	36.7 %
		No influence	70	36.7 %
	Fear of delivering alone	Low	170	28.3 %
		High	430	71.7 %
	Stress about the future	Low to average	333	55.5 %
		High	267	44.5 %
Physical exercise	Weekly exercise before the restrictions (*N* = 460)	More than 2 h	268	58.3 %
		Less than 2 h	192	41.7 %
	Weekly exercise after the restrictions (*N* = 359)	More than 2 h	139	38.8 %
		Less than 2 h	220	61.3 %
	Changes pre vs. post lock-down of weekly minutes of physical exercise (*N* = 503)	As before	88	17.5 %
		More than before	104	20.7 %
		Less than before	311	61.8 %
	Restrictions gave you the chance to exercise more	Yes	86	14.3 %
		No	514	85.7 %
	Not walking outside: influence on your wellbeing ^a^	Low	115	80.8 %
		High	484	19.2 %
Dietary habits	Restrictions gave you the chance to eat more healthily ^a^	Yes	266	44.3 %
		No	334	55.7 %
Access to care	Participation in online pre-birth course ^a^	Currently participating	149	24.9 %
		About to start	10	1.7 %
		Not participating	439	73.4 %
	Access to emergency room	Gave up to go to ER	72	12.0 %
		No events	528	88.0 %
	How did you solve the problem (*N* = 72)	Phone call with Gynecologist	56	77.8 %
		Phone call with midwife	24	33.3 %
		Visit to private Gynecologist	35	48.6 %
		Did not resolve	5	6.9 %
	did you skip any planned check up?	Yes	159	26.5 %
		No	441	73.5 %
	did you skip any planned test or vaccination? ^a^	Yes	112	18.8 %
		No	483	81.2 %

^a^ Presence of missing data for these variables. Percentages were calculated on total of respondents: Influence of partner at home (*N* = 562), Not walking influences on your wellbeing (*N* = 599), Participation in online pre-birth course (*N* = 598), Have you skipped any planned test or vaccination (*N* = 595)

Concerning dietary habits, according to 44.3 % of women, social restrictions gave them the chance to eat more healthily.

Regarding access to health care services, only 24.9 % of women in our sample was attending an online pre-birth course and 12 % of them avoided access to an ob-gyn emergency department for fear of contagion, preferring phone contact with a gynecologist or a midwife. Finally, 26.5 % of our sample skipped some planned check-up and 18.8 % skipped planned tests or vaccinations.

### The experience of delivering during lockdown for new mothers

Table 3 describes the experience of delivery and baby management during lockdown. The overall response rate for these items was > 99 %. The mean gestational age at the time of delivery was 39.4 ± 1.3 weeks (range 36–42 weeks). 92.4 % of the partners had the possibility to be present during labor. 75.3 % of women declared they were afraid of giving birth during the COVID-19 pandemic. They reported that the delivery experience was as they expected in 50.8 % of cases, better than expected in 36.2 %.

**Table 3:** The experience of delivering during the lockdown among new mothers

	Parameter	Category	*N* = 139	%
**Delivery and obstetrics care**	Delivery mode	Vaginal Eutocic	96	69.1 %
		Vaginal dystonic	22	15.8 %
		Caesarean section	21	15.1 %
	Presence of partner during delivery (*N* = 118) ^b^	Yes	109	92.4 %
		No	9	15.8 %
	Worried about receiving lower quality assistance because of the pandemic ^a^	Yes	60	44.1 %
		No	76	55.9 %
	Reality versus expectations for you ^a^	As expected	67	50.7 %
		Better	50	36.2 %
		Worst	18	13.0 %
**Neonatal care and breastfeeding**	Required neonatal intensive care	Yes	15	10.8 %
		No	124	89.2 %
	Worried about receiving lower quality neonatal assistance ^a^	Yes	36	26.0 %
		No	102	74.0 %
	Reality versus expectations for your baby	As expected	83	59.7 %
		Better	42	30.2 %
		Worst	14	10.1 %
	Influence of restrictions on neonatal management	No influence	43	30.9 %
		Negative influence	85	61.2 %
		Positive influence	11	7.9 %
	Breastfeeding during hospital stay	Yes	131	94.0 %
		No	8	6.0 %
	Type of nutrition	Exclusive breastfeeding	98	70.5 %
		Formula feeding	38	27.3 %
		Human donor milk	3	2.2 %
	Continued breastfeeding after discharge	Yes	132	95.0 %
		No	7	5.0 %
	Still breastfeeding at the time of survey	Yes	126	90.6 %
		No	13	9.4 %
	Influence of restrictions on breastfeeding	No influence	78	56.1 %
		Negative influence	51	36.7 %
		Positive influence	10	7.2 %
	Who supported you for breastfeeding after the discharge (more than one choice available)	Midwife ^c^	36	55.4 % ^d^
		Partner	27	41.5 % ^d^
		Relative or friend	18	27.7 % ^d^
		Pediatrician	4	6.1 % ^d^
		No support	74	53.2 %

^a^ Presence of missing data for these variables. Percentages were calculated on total of respondents: Afraid of receiving worst assistance for the pandemic (*N* = 136), Reality versus expectations (*N* = 135), Afraid of receiving a worse neonatal assistance (*N* = 138); ^b^Percentages were calculated on total of non-caesarean deliveries (*N* = 118); ^c^ This is the total of respondents declared any type of assistance in breastfeeding after discharge by midwife (*n* = 36). Among these, 20 new mothers claimed that they had assistance by a private midwife, another 20 by midwife of public surgery, and/or 5 by midwife of the hospital where they delivered. Total number is bigger than the frequency shown in Table 3 (*n* = 36) because this question allowed more than one choice. ^d^ Percentages were calculated on the total of women who claimed to have received support for breastfeeding after the discharge (*N* = 65)

Overall, the restrictive measures had a negative impact on baby management for 61.1 % of the new mothers, and no impact for 28.1 %. Most of the respondents reported to have breastfed their babies during the hospital stay (94.2 %) and about two-thirds of them started breastfeeding within the first two hours after delivery (64.7 %). During the hospital stay, 70.5 % of the babies were exclusively breastfed, 27.3 % received formula feeding, and 2.2 % received human donor milk. The majority of the new mothers declared to have continued to breastfeed their babies when discharged at home (95.0 %), and most of them (91.7 %) stated they were still breastfeeding the babies at the time of the survey, reporting exclusive breastfeeding in 85.0 % of cases and mixed with formula feeding in 5.8 %.

No impact of restrictive measures on breastfeeding was reported by 56.1 % of the new mothers, a negative impact by 36.7 %, and a positive one by 7.2 %. After hospital discharge, the respondents reported having received no support for breastfeeding in 53.2 % of cases, whereas 55.4 % of women who received support claimed that this came from a midwife and 41.5 % from their partner. Only a few of the respondents (6.1 %) reported having had support from a pediatrician for breastfeeding during the first period after discharge. In our sample, only 3 women (2.2 %) reported to have had confirmed SARS-CoV-2 infection at the time of delivery: all of them were separated from their newborns maintaining the possibility to feed them with expressed breast milk. Regarding the rest of the respondents, it was not known if they were not tested or tested negative, because it was not inquired in the survey.

### Adjusted analysis for pregnant women

Table 4 shows χ2 and prevalence across independent variables and three crucial outcomes in the pregnant women group: PH4 scores from moderate to severe, difficulties in healthy eating and reduction in physical exercise.

**Table 4:** Changes in mental wellness and lifestyles during lockdown by socio-demographic characteristics, housing and living conditions

		Anxiety and depression				Difficulties in healthy eating				Reduction in physical exercise			
		PHQ-4 Moderate to Severe N (%)		Chi2 *p*-value	PR (95% CI) ^a^	Yes N (%)		Chi2 *p*-value	PR (95% CI) ^a^	Yes N (%)		Chi2 *p*-value	PR (95% CI) ^a^
Age	Less than 34	236	62.3%		1	167	44.1%		1	200	62.5%		1
	From 35	141	63.8%	0.71	1.02 (0.83 - 1.26)	99	44.8%	0.86	1.01 (0.79-1.30)	111	60.7%	0.68	0.97 (0.77-1.22)
Education	Less than Bachelor	158	68.7%		1	127	55.2%		1	112	63.3%		1
	More than Bachelor	218	59.1%	0.02	0.86 (0.70-1.05)	138	37.4%	<0.001	0.7 (0.53-0.86)	198	60.9%	0.61	0.96 (0.76-1.21)
Satisfaction with economical resources	Not much adeguate	224	71.8%		1	153	49.0%		1	162	64.8%		1
	Very adeguate	150	52.6%	<0.001	0.73 (0.60-0.90)	113	39.6%	0.02	0.80 (0.63-1.03)	148	59.2%	0.20	0.91 (0.73-1.14)
Satisfaction with your home	Not much	172	74.4%		1	118	50.9%		1	128	65.5%		1
	Very much	205	55.7%	<0.001	0.75 (0.61-0.92)	148	40.2%	0.01	0.79 (0.62-1.01)	183	59.5%	0.20	0.92 (0.73-1.17)
Partner supporting	No	78	77.2%		1	60	59.4%		1	52	66.7%		1
	Yes	299	59.9%	0.001	0.77 (0.60-0.99)	206	41.3%	0.001	0.69 (0.52-0.93)	259	60.9%	0.34	0.91 (0.68-1.23)
Contacts with other people	No	239	63.0%		1	154	41.0%		1	197	62.1%		1
	Yes	138	61.6%	0.63	0.96 (0.78-1.19)	112	50.0%	0.03	1.22 (0.96-1.56)	114	61.3%	0.85	0.98 (0.78-1.24)
Availability of free time	As before	71	65.7%		1	45	41.7%		1	55	63.2%		1
	Less than before	102	65.4%		0.99 (0.73-1.34)	77	49.4%		1.18 (0.82-1.74)	86	69.9%		1.10 (0.79-1.55)
	More than before	203	60.8%	0.49	0.92 (0.70-1.21)	143	42.8%	0.33	1.02 (0.73-1.44)	168	57.7%	0.06	0.91 (0.67-1.24)
Other children at home	No	250	61.6%		1	165	40.6%		1	217	59.6%		1
	Yes	127	65.5%	0.35	1.06 (0.86-1.31)	101	52.1%	0.01	1.28 (1.00-1.64)	94	67.6%	0.10	1.13 (0.89-1.44)
Trimester of pregnancy	First	38	70.4%		1	16	29.6%		1	38	90.5%		1
	Second	112	56.6%		0.80 (0.55-1.16)	76	38.4%		1.29 (0.75-2.22)	99	58.9%		0.64 (0.44-0.93)
	Third	223	64.8%	0.07	0.92 (0.51-1.30)	172	50.0%	0.002	1.69 (1.01-2.81)	172	59.9%	<0.001	0.66 (0.47-0.94)

^a^ Adjusted Prevalence Ratios calculated by Poisson regression with their 95% Confidence Intervals (CI)

Poisson analysis showed that women with partner support during pregnancy and satisfied with economic and house resources were less likely to report higher anxiety and depression scores (respectively − 23 %, -27 %, and − 25 %) compared to women without partner support and not satisfied with economic and house resources.

While for most women restrictions gave them the chance to eat more healthily, 20.2 % of women reported difficulties in healthy eating. In particular, women with partner support declared less difficulty in healthy eating (-31 %). Likewise, higher educational attainment was also associated with less difficulty in healthy eating (PR 0.70, 95 % CI 0.53–0.86).

Lastly, during lockdown there was a significant reduction in physical activity, but this data is transversal to all respondents and there are no significant differences between groups, except for the trimester of pregnancy: women in the second and third trimesters were less likely to have reduced levels of physical activity during lockdown (− 36 and − 34 %, respectively) in comparison to women in the first trimester.

## Discussion

This study describes how pregnant women managed to cope with lockdown in Italy. We found a high score for anxiety and depression, although it cannot be compared to the same score on the same population before the pandemic. Our survey also suggests that lockdown made it more difficult for pregnant women to exercise for 150 min per week in accordance with the ACOG guidelines [14], and we can assume that a reduction in physical exercise will affect the quality of life of pregnant women, as demonstrated in previous studies [15]. On the other hand, it seems that staying at home facilitated the approach to healthy eating for the group with partner support and a higher socio-economic status. This results deserve further investigation and they could represent a starting point to develop new strategies for public health.

Most women hope for a labor and birth experience that enables them to use their physical and psychosocial capacities to labor and give birth to a healthy baby in a clinically, culturally, and psychologically safe environment with continuity of practical and emotional support from a birth companion, and with kind, sensitive clinical staff, who provide reassurance and technical competency. Among women who delivered during the pandemic, although three-fourths of the respondents declared to be afraid of giving birth during such a complex period, the overall experience was as expected or better than expected for 87 % of the respondents. Despite more than half of the new mothers reported a negative influence on baby management and more than one-third of them reported a negative influence on their breastfeeding experience, the breastfeeding rate is consistent or even higher than before the pandemic [16], suggesting a slight discrepancy between expectations/perceptions and actual facts, probably due to the anxiety and depression characteristics found in our sample. Exclusive breastfeeding rates in Italy ranged from 57 to 77 % at discharge and 36 to 48 % after 3 months [16], while 95 % of our sample declared to perform it at discharge and 90.6 % of them were still breastfeeding at the time of the survey. More than half of the new mothers received no support for breastfeeding after hospital discharge; however, almost all respondents continued to breastfeed their babies when discharged at home. It could be inferred that, in the impossibility to rely on external support, new mothers have empowered their internal resources with satisfying results.

The most accurate comparison we could make about the delivery and postpartum experience is with the Italian data of CeDAP published in 2016 by the Italian Ministry of Health. (http://www.salute.gov.it/imgs/C_17_pubblicazioni_2881_allegato.pdf). The median age of women giving birth in Italy in 2016 was 33 years, consistent with our sample. Of women giving birth in 2016 in Italy, 27.8 % were graduated, while in our sample the women with a bachelor were 61.8 % in the pregnant group and 73.4 % of the new mothers. In the report of 2016, it results that 55.3 % of women giving birth was in-work, while in our sample 75.7 and 78.3 % were in-work. This must be considered as a bias, as discussed later on. In 2016, the partner accompanied the woman during labor in 92.2 % of cases, comparable to the rate of 92.4 % in our sample. Furthermore, the cesarean section rate in our sample was just 15.1 %, much lower than the 33.7 % in 2016. However, we cannot speculate if this difference is given by the pandemic or it is just a selection bias.

The high level of anxiety and depression we found is consistent with other studies [7, 8]. The prevalence in the first trimester is confirmed [8] while, differently from another study [7], in our survey this data was not correlated with age, primiparity, and living area. The correlation with economic difficulties and lower education is consistent with the literature [15], and some studies suggested that COVID-19 pandemic may even worsen social inequality [17].

Regarding the fact that a reduction of face-to-face visits could have occurred during the restrictions, a recent survey showed that patients are actually open to alternative models of prenatal care, including remote monitoring [18]. Future surveys could be done to determine if such changes would be judged positively. According to a Cochrane review [19], communicating the results of medical investigations by mobile phone messaging may make little or no difference to women’s anxiety overall or in women with positive test results, but may reduce anxiety in women with negative test results. We cannot exclude that this method will be more largely implemented in future times, after the COVID-19 emergency and the lessons it gave us about face-to-face contact.

The high prevalence of anxiety and depressive symptoms in pregnant women and new mothers should be a public health issue, and screening for perinatal depression and anxiety should be considered during a pandemic. Under the circumstances of social distancing and isolation, psychological hotlines and online counseling would be a smart strategy to manage perinatal mental illness. The same strategy would be useful to help new mothers with baby management. Healthcare professionals should also ensure patients feel supported by continuing their routine prenatal care through tele-medicine visits [20]. Clinicians might also consider recommending and encouraging “home” physical exercise, especially in women in the first trimester, who might be most worried about the sudden change of their lives.

Isolation, increased stress, and sedentary lifestyle in pregnancy can also lead to adverse pregnancy outcomes, such as preterm birth, gestational diabetes, and low birth weight [21, 22]. This survey represents a baseline questionnaire for those women who gave consent to be contacted, and they will be followed up as a cohort to identify possible complications. In a further part of our project, we are going to describe in greater detail how lockdown may influence neonatal outcomes.

The first limitation of the present study is related to nonrandom sampling: women are enrolled by newspaper advertisements, social media, and with the snowball method; the completeness check process was not exhaustive. This enrollment method accounts for the possible bias represented by the high number of respondents from Northern Italy, as the research group was based in Northern Italy and the spreading of the link to the survey by social media would have been greater and faster where the research was conceptualized. Moreover, the enrollment by social media and dedicated newspapers implies the intrinsic limit that the most wealthy and educated segments of the population might be more easily reached by the invitation to answer the survey, as suggested by the high rate of respondents with at least a bachelor’s degree and reporting their income was highly sufficient to their needs. A second limitation is the lack of a validated questionnaire designed to capture such a delicate and unique moment. Third, depressive and anxiety symptoms were assessed using a short scale relying on self-reported measures and not providing a diagnosis. Finally, a potential bias may de that a propensity score analysis was not performed and we did not account for weighting of the items. Despite these limitations, this is the first study to assess some aspects of the lifestyle of pregnant women and new mothers during lockdown in Italy. Besides, the web-based method is a strength because it gave us the opportunity to interview a geographically dislocated population during a short time in the lockdown period.

Given the unicity of the SARS-CoV2 pandemic, we tried to give an overview of the experience of Italian pregnant women and new mothers during lockdown. Next steps will be to incorporate those findings in political choices. The WHO Executive Board recognizes the need to include women in decision making for outbreak preparedness and response, however there is still inadequate women representation in national and global COVID-19 policy spaces [23]. It is also important that health professionals commit themselves to help pregnant women and new mothers to overcome these difficult times.

## Conclusions

In conclusion, we found a high prevalence of anxiety and depressive symptoms in pregnant women and new mothers, which should be a public health issue. Almost two-thirds of the respondents reported a reduction of physical activity during lockdown; therefore, clinicians might consider recommending and encouraging “home” physical exercise. On the other hand, about half of the sample improved their approach towards healthy eating and a very high breastfeeding rate was reported soon after birth: these data are an interesting starting point to develop new strategies for public health.

## Supplementary Information

**Additional file 1:** Supplementary Material_questionnaire: survey_rev: English version of the questionnaire

**Additional file 2:** Supplementary Material_questionnaireITA: survey_revITA: original Italian version of the questionnaire

## Data Availability

The datasets generated and/or analysed during the current study are not publicly available due to privacy reasons but are available from the corresponding author on reasonable request.

## References

[1] Kraemer MUG, Yang C-H, Gutierrez B, Wu C-H, Klein B, Pigott DM (2020). The effect of human mobility and control measures on the COVID-19 epidemic in China. Science.

[2] Rasmussen SA, Smulian JC, Lednicky JA, Wen TS, Jamieson DJ (2020). Coronavirus Disease 2019 (COVID-19) and pregnancy: what obstetricians need to know. Am J Obstet Gynecol.

[3] Rasmussen SA, Jamieson DJ (2020). Coronavirus Disease 2019 (COVID-19) and Pregnancy: Responding to a Rapidly Evolving Situation. Obstet Gynecol.

[4] Wenham C, Smith J, Morgan R (2020). COVID-19: the gendered impacts of the outbreak. The Lancet.

[5] Della Gatta AN, Rizzo R, Pilu G, Simonazzi G. COVID19 during pregnancy: a systematic review of reported cases. Am J Obstet Gynecol. 2020;S0002937820304385. 10.1016/j.ajog.2020.04.013 PMC7165087 32311350

[6] Du L, Gu YB, Cui MQ (2020). [Investigation on demands for antenatal care services among 2 002 pregnant women during the epidemic of COVID-19 in Shanghai]. Zhonghua fu Chan ke za zhi.

[7] Wu Y, Zhang C, Liu H, Duan C, Li C, Fan J, et al. Perinatal depressive and anxiety symptoms of pregnant women along with COVID-19 outbreak in China. Am J Obstet Gynecol. 2020;S0002937820305342. 10.1016/j.ajog.2020.05.009 PMC7211756 32437665

[8] Saccone G, Florio A, Aiello F, Venturella R, De Angelis MC, Locci M, et al. Psychological Impact of COVID-19 in pregnant women. Am J Obstet Gynecol. 2020;S0002937820305275. 10.1016/j.ajog.2020.05.003 PMC7204688 32387321

[9] Wang C, Pan R, Wan X, Tan Y, Xu L, Ho CS (2020). Immediate Psychological Responses and Associated Factors during the Initial Stage of the 2019 Coronavirus Disease (COVID-19) Epidemic among the General Population in China. Int J Environ Res Public Health.

[10] von Elm E, Altman DG, Egger M, Pocock SJ, Gøtzsche PC, Vandenbroucke JP (2014). The Strengthening the Reporting of Observational Studies in Epidemiology (STROBE) Statement: Guidelines for reporting observational studies. Int J Surg.

[11] Eysenbach G (2004). Improving the Quality of Web Surveys: The Checklist for Reporting Results of Internet E-Surveys (CHERRIES). J Med Internet Res.

[12] Marcano Belisario JS, Jamsek J, Huckvale K, O’Donoghue J, Morrison CP, Car J. Comparison of self-administered survey questionnaire responses collected using mobile apps versus other methods. Cochrane Database Syst Rev. 2015;(7):MR000042. 10.1002/14651858.MR000042.pub2 PMC8152947 26212714

[13] Kroenke K, Spitzer RL, Williams JBW, Lowe B (2009). An Ultra-Brief Screening Scale for Anxiety and Depression: The PHQ-4. Psychosomatics.

[14] Physical activity and exercise during pregnancy and the postpartum period (2020). ACOG Committee Opinion No. 804. American College of Obstetricians and Gynecologists. Obstet Gynecol.

[15] Lagadec N, Steinecker M, Kapassi A, Magnier AM, Chastang J, Robert S (2018). Factors influencing the quality of life of pregnant women: a systematic review. BMC Pregnancy Childbirth.

[16] Davanzo R, Romagnoli C, Corsello G (2015). Position Statement on Breastfeeding from the Italian Pediatric Societies. Ital J Pediatr.

[17] Onwuzurike C, Meadows AR, Nour NM (2020). Examining Inequities Associated With Changes in Obstetric and Gynecologic Care Delivery During the Coronavirus Disease 2019 (COVID-19) Pandemic. Obstet Gynecol.

[18] Peahl AF, Novara A, Heisler M, Dalton VK, Moniz MH, Smith RD (2020). Patient Preferences for Prenatal and Postpartum Care Delivery: A Survey of Postpartum Women. Obstet Gynecol.

[19] Gurol-Urganci I, de Jongh T, Vodopivec-Jamsek V, Car J, Atun R. Mobile phone messaging for communicating results of medical investigations. Cochrane Database Syst Rev. 2012 Jun 13;2012(6):CD007456. 10.1002/14651858.CD007456.pub2 PMC6486139 22696369

[20] Jago CA, Singh SS, Moretti F. Coronavirus Disease 2019 (COVID-19) and Pregnancy: Combating Isolation to Improve Outcomes. Obstet Gynecol. 2020 Jul;136(1):33 – 6. 10.1097/AOG.0000000000003946 32384386

[21] Elsenbruch S, Benson S, Rücke M, Rose M, Dudenhausen J, Pincus-Knackstedt MK (2007). Social support during pregnancy: effects on maternal depressive symptoms, smoking and pregnancy outcome. Hum Reprod.

[22] Shapiro GD, Fraser WD, Frasch MG, Séguin JR. Psychosocial stress in pregnancy and preterm birth: associations and mechanisms. J Perinat Med. 2013 Nov;41(6):631–45. 10.1515/jpm-2012-0295 PMC5179252 24216160

[23] Boniol M, McIsaac M, Xu L, Wuliji T, Diallo K, et al. Gender equity in the health workforce: analysis of 104 countries. World Health Organization. 2019. https://apps.who.int/iris/handle/10665/311314. License: CC BY-NC-SA 3.0 IGO.

