# Supplementary material for: The perception of Italian pregnant women and new mothers about their psychological wellbeing, lifestyle, delivery, and neonatal management experience during the COVID-19 pandemic lockdown: a web-based survey
Source: BMC Pregnancy Childbirth. 2021 Jul 1;21:473. doi: 10.1186/s12884-021-03904-4 (PMC8246432; doi:10.1186/s12884-021-03904-4)
Supplement: Supplementary file 1 — Additional file 1: Supplementary Material_questionnaire: survey_rev: English version of the questionnaire [file 12884_2021_3904_MOESM1_ESM.docx]

# Coping with social distancing: a cross sectional survey among Italian pregnant women and new-mothers during the COVID-19 pandemic lockdown

# QUESTIONNAIRE

## General section

### 1.1 Demographic data

**Year of birth**

[specify the number]

**Nationality**

[specify nationality]

**Region**

[specify Italian region of your residence]

**Educational degree**

None

Primary school

Middle school

High School

University Graduation

Postgraduate

**Does your partner live with you at the moment?**

Yes and he doesn’t go to work

Yes and he regularly goes to work

Yes and he goes to work less frequently

Yes, he is isolated because he has Covid like symptoms

Yes, he is isolated because he was diagnosed with Covid

No, he does not live with me

No, he is hospitalized

No, he is isolated somewhere else

**Do you have children living with you?**

Yes

No

**If yes, please tell us the number of children**

[specify the number]

**Does anyone else live with you, other than your partner and your children?**

**Do you have contacts with other people living close to your house?**

No

Yes, seldom

Yes, often

Yes, every day.

**How often do you follow (accurately are you following) the restrictive measures given by the Government about movements and face-to face contacts?**

always

often

sometimes

seldom

never

**Are you currently quarantined by the Authorities?**

No

Yes because I have Covid-like Symptoms

Yes because I was diagnosed with Covid

No, I am hospitalized

**Where do you live?**

Downtown of a city

Suburbs of a city

Small/ medium town in the countryside

Small/medium town by the sea

Isolated house (or in a very small village with a few hundred inhabitants)

**Is the house where you are living adequate to your needs?**

Not at all

not really

quite

very

extremely

**How big is the house you are living in?**

Less than 50 mq

From 50 to 70 mq

From 70 to 100 mq

More than 100 mq

**Are there any outdoor spaces?**

No

A little balcony

One or more terraces

Garden

**Are the electronic devices that you have adequate to your needs?**

Not at all

not really

quite

very

extremely

**Is the money you have adequate to your needs?**

Not at all

not really

quite

very

extremely

### 1.2 Psychological aspects

**Do you agree with these sentences (1-not at all true; 5-very true)?**

I can always manage to solve difficult problems if I try hard enough.

Thanks to my resourcefulness, I know how to handle unforeseen situations

I can solve most problems if I invest the necessary effort.

I can remain calm when facing difficulties because I can rely on my coping abilities.

**Are you satisfied with your life?**

Extremely

Very

Quite

A little

Not at all

**Over the last two weeks, how often have you been bothered by the following problems? (0- not at all; 3 nearly every day)**

Feeling nervous, anxious or on edge

Not being able to stop or control worrying

Feeling down, depressed or hopeless

Little interest or pleasure in doing things

**Who are the people that you feel are supporting you the most during this period (more answers possible)?**

Partner

Mother

Father

Sisters/Brothers

Friends

Mother/father in law

Other relatives

Midwife

Gynecologist

Other health workers

Women I know who are pregnant or have just delivered

Facebook

Websites

### 1.3 Job related questions

**Are you currently**

Unoccupied

Student

Housewife

In-work

**What is your job**

Employee

Executive

Freelance

Worker

Farmer

Saleswoman

Teacher

Nurse

Medical doctor

Midwife

Other health workers

Researcher

Educator

Police officer

Other _____

**regarding my job during these days**I am at home/smart working

I regularly go to my workplace

I go to my workplace with less frequency

I work both from home and from my workplace

My job has been suspended

I got fired

I am on maternity leave

**Do you think the governmental restrictions related to COVID19 pandemic had an impact on your approach to work?**

Yes, a positive impact

Yes, a negative impact

No

## Pregnant women section

### 2.1 Obstetrical history

**Is this your first pregnancy**

Yes

No

**At which week of pregnancy are you currently?**

[specify the number]

**Which is the expected delivery day?**

[specify the date]

**Is it a singleton or twin pregnancy?**

Singleton

Twin

**Did this pregnancy come spontaneously or after reproduction techniques?**

Spontaneously

After reproduction techniques

**How long did you look for this pregnancy?**

More than one year

Less than one year

Less than three months

It was not looked for

**Have you ever had a miscarriage? (voluntary interruptions are not included)**

Please specify how many

**IF THIS IS NOT YOUR FIRST PREGNANCY**

**At how many gestational weeks were your children born?**

[specify the number]

**What was their weight at birth?**

[specify the number in kg]

**Did you have any medical issues in your previous pregnancy? *(more than one answer is possible*)**

Gestational diabetes

Hypertensive disorder of pregnancy

Maternal infection

Urgent cesarean section

Fetal sufferance

Placental abruption

IUGR

Other

**Did you have any illness before the pregnancies? *(more than one answer is possible)***

Diabetes

Hypertension

Autoimmune pathologies

Coagulation problems

Anxiety or depression

Thyroid disease

Other

**Are you a smoker?**

No

Yes

I quit during this pregnancy

**How much do you weigh?**

[specify the number in kg]

**Which was your weight before the pregnancy**

[specify the number in kg]

**How tall are you?**

[specify the number in cm]

**So far, was this pregnancy diagnosed with any of the following? *(more than one answer is possible*)**

- risk of miscarriage
- risk of premature delivery
- pregnancy related hypertensive disorder
- Gestational diabetes
- Vaginal infections
- COVID 19 infection
- IUGR
- other

### 2.2 Spare time

**Since the governmental restrictions related to COVID19 pandemic are in force has your quantity of spare time changed?**

A lot less than usual

A bit less than usual

As usual

A bit more than usual

A lot more than usual

**How much of your spare time is actually yours (and not invested in homekeeping or childcare?)**

A lot less than usual

A bit less than usual

As usual

A bit more than usual

A lot more than usual

**Do you believe having more spare time has an impact on your pregnancy?**

No

Yes, a positive one

Yes, a negative one

### 2.3 Physical exercise

**Not considering the pandemic, do you consider yourself a sporty person?**

Extremely

Very

Quite

A little

Not at all

**How much time did you invest on physical exercise before the restrictions were in force (including Yoga, brisk walking, fitness, etc..)?**

more than 5 hours per week

between 3 and 5 hours per week

between 2 and 3 hours per week

between half an hour and 2 hours per week

less than half an hour

**How much time did you invest on physical exercise in the last two weeks (including Yoga, brisk walking, fitness, etc..)?**

more than 5 hours per week

between 3 and 5 hours per week

between 2 and 3 hours per week

between half an hour and 2 hours per week

less than half an hour

**Do you think becoming pregnant had an impact on your approach to physical exercise?**

No

Yes, a positive one

Yes, a negative one

**Do you think the governmental restrictions related to COVID19 pandemic had an impact on your approach to physical exercise?**

No

Yes, a positive one

Yes, a negative one

### 2.4 Eating

**Not considering the pandemic, how often do you eat healthily?**

Always

Often

Sometimes

Seldom

Never

**Since the beginning of the lockdown, how often have you had difficulties eating healthily?**

Always

Often

Sometimes

Seldom

Never

**Do you think becoming pregnant had an impact on your approach to healthy eating?**

No

Yes, a positive one

Yes, a negative one

**Do you think the governmental restrictions related to COVID19 pandemic had an impact on your approach to healthy eating?**

No

Yes, a positive one

Yes, a negative one

2.5 *Pros* and *cons*

**If your partner is more often at home, does this fact have an influence on your pregnancy?**

No

Yes, a positive one

Yes, a negative one

**If you have other children, having them at home has an influence on your pregnancy?**

No

Yes, a positive one

Yes, a negative one

**If your job is suspended, does this fact have an influence on your pregnancy?**

No

Yes, a positive one

Yes, a negative one

**How much do these statements influence your pregnancy? (1 not much – 5 very much)**

Not being able to walk outside

Not meeting friends and relatives

Not attending pre-birth courses

### 2.6 Access to care

**During the lockdown, have you had a health emergency but you did not go to the emergency room for fear of the pandemic?**

Yes

No

**How did you solve the problem?**

Phone call with gynecologist

Gynecological private visit

Midwife private visit

Public ambulatory visit

General practitioner

Pharmacist

Help of relatives

Found the answer online

Watchful waiting

The problem is not solved

**Are you participating in online pre-birth courses?**

Yes

No

**During the lockdown have you skipped any scheduled checks?**

Yes

No

**During the lockdown have you skipped any test or vaccination?**

Yes

No

**Does the pandemic generate anxiety about the future?**

**How often does this pandemic generate anxiety about the future?**

Always

Often

Sometimes

Seldom

Never

**Do you have fears about your delivery?**

Many

Numerous

Some

A few

None

**Tell us your main worries?**

_____

**How worried are you that your partner will not be able to accompany you?**

Very much

Much

Enough

Little

Not at all

**Are you afraid of being left alone with your baby when he/she will be born?**

Very much

Much

Enough

Little

Not at all

**Do you suggest any intervention that could help pregnant women in these difficult times?**

**…..**

**Which of those interventions could also be useful after the pandemic ?**

**…..**

## New-mothers section

### 3.1 Obstetrical history

**Have you participated in a pre-birth course?**

Yes

No

**Have you participated in an online pre-birth course?**

Yes

No

**When did you give birth?**

[specify the date]

**In which city?**

[specify the City]

**Was it your first pregnancy?**

Yes

No

**IF THIS IS NOT YOUR FIRST PREGNANCY**

**At how many gestational weeks were your other children born?**

[specify the number]

**What was their weight at birth?**

[specify the number in kg]

**How many months did you breast feed them?**

[specify the number]

**At how many weeks did you give birth during this pregnancy?**

[specify the number]

**Was it a singleton or a twin pregnancy?**

Singleton

Twin

**How did you deliver?**

Eutocic vaginal delivery

Inducted vaginal delivery

Dystocic spontaneous vaginal delivery

Dystocic induced vaginal delivery

Cesarean section

**Did you recur to epidural analgesia?**

Yes

No

**Who accompanied you in the delivery room? *(more than one answer is possible*)**

Midwife

Gynecologist

Partner

Friend or relative

**Did you have any illness before the pregnancy? *(more than one answer is possible)***

Diabetes

Hypertension

Autoimmune pathologies

Coagulation problems

Anxiety or depression

Thyroid disease

Other

**Have you had any medical issues during this pregnancy? *(more than one answer is possible*)**

Gestational diabetes

Pregnancy-related hypertensive disorder

Maternal infection

Urgent cesarean section

Fetal sufferance

Placental abruption

IUGR

COVID 19

Other

**How many days did the hospitalization last after the delivery?**

[specify the number]

**Did you have any complications after the delivery?**

[specify complications]

**Did your baby require any intensive care?**

Yes

No

**For which problem?**

[specify the problem]

**How many days was he/she hospitalized?**

[specify the number]

### 3.2 Breastfeeding

**After the delivery did you breastfeed?**

Yes, within the first two hours after the delivery

Yes, during the first day

Yes, in the following days

No, I chose not to

No, I had difficulties

**During the hospitalization, what did your baby eat?**

My breastfed milk

my breastfed milk and formula milk

Human donor milk

Only formula milk

Other

**Who helped you in the hospital? *(more than one answer is possible)***

Midwife

Nurse

Gynecologist

Neonatologist

None

**At the time of discharge, did you feel able to breastfeed?**

Yes

No

**After the discharge, did you breastfeed your baby?**

Yes

No

**Are you still breastfeeding your baby?**

Yes

No

**After the discharge, who helped you with breastfeeding? (more than one answer is possible)**

Nobody

Partner

Mother

Father

Brother/sister

Mother-in-law

Friend/other relative

Midwife of the hospital

Midwife of the national health service

Private midwife

Gynecologist

Pediatrician

Other

**Do you think the lockdown had an influence on your breastfeeding?**

No

Yes, a positive one

Yes, a negative one

**Why?**

[specify reason]

### 3.3 Baby management

**Was your baby visited by a neonatologist during hospitalization?**

Yes, everyday

Yes, at birth

Yes, before discharge

No

I don’t know

**If you were Covid positive, were you separated from your baby?**

Yes

No

**If you were Covid positive, was it possible for you to give your milk to the baby?**

Yes

No

**During hospitalization did you become capable of managing your baby’s hygiene ?**

Yes

No

**During hospitalization did you learn how to make umbilical dressings to your baby?**

Yes

No

**After the discharge, who helped you with hygiene and dressings of your baby? *(more than one is possible)***

Nobody

Partner

Mother

Father

Brother/sister

Mother-in-law

Friend/other relative

Midwife of the hospital

Midwife of the public service

Private midwife

Gynecologist

Pediatrician

Other

**In the week after the discharge, has your baby been visited? *(more than one is possible)***

No

Yes, in the hospital

Yes, in the public surgery

Yes, at the pediatrician’s

Yes, in the emergency room

**Do you think the lockdown had an influence on your baby management?**

No

Yes, a positive one

Yes, a negative one

### 3.4 Psychological aspects

**How worried were you of giving birth during a pandemic?**

Very much

Much

Enough

Little

Not at all

**Were you afraid of receiving less assistance?**

Yes

No

**Compared to your expectations about the health assistance you received reality was**

Better

As expected

Worst

**Were you afraid your baby would receive less care?**

Yes

No

**Compared to your expectations about the assistance given to your baby, reality was**

Better

As expected

Worst

**Do you think the lockdown had an impact on your mood at home, once discharged?**

Yes

No

**How?**

[specify impact on your mood]

**If there had not been a lockdown, once discharged:**

I would have received more help

I would have received less help

Nothing would have changed

**How capable did you feel of managing your baby?**

Very much

Much

Enough

Little

Not at all
