# Supplementary material for: The perception of Italian pregnant women and new mothers about their psychological wellbeing, lifestyle, delivery, and neonatal management experience during the COVID-19 pandemic lockdown: a web-based survey
Source: BMC Pregnancy Childbirth. 2021 Jul 1;21:473. doi: 10.1186/s12884-021-03904-4 (PMC8246432; doi:10.1186/s12884-021-03904-4)
Supplement: Supplementary file 2 — Additional file 2: Supplementary Material_questionnaireITA: survey_revITA: original Italian version of the questionnaire [file 12884_2021_3904_MOESM2_ESM.docx]

# #mammeacasa

# L’impatto delle misure restrittive da decreto #iorestoacasa su gravidanza e puerperio

QUESTIONARIO

1. Domande introduttive

1.1 Dati demografici

**Indica il tuo anno di nascita**

….

**Qual è la tua nazionalità?**

**….**

**Qual è la regione in cui abiti in questo momento?**

….

**Indica il tuo titolo di studio**

Nessuno

Licenza elementare

Licenza di scuola media inferiore

Licenza di scuola superiore

Laurea

Post laurea

**In questo momento il tuo partner vive con te?**

Sì e rispetta le restrizioni totali previste dal decreto #iorestoacasa

Sì ma continua a recarsi al lavoro come prima

Sì ma si reca al lavoro come meno frequenza di prima

Sì ed è in isolamento domiciliare fiduciario in quanto presenta sintomi suggestivi per CoVID-19

Sì ed è in isolamento domiciliare fiduciario in quanto ha un tampone positivo per CoVID-19

No, non vive con me

No, è attualmente ricoverato

**Hai figli che vivevano già con te, prima di questa gravidanza?**

Sì

No

**Se sì, quanti?**

[numero]

**Qualcun altro vive con te, oltre all'eventuale partner ed eventuali figli?**

**Hai contatti con altri familiari o amici che vivono nei pressi del tuo domicilio?**

No

Sì, saltuari

Sì, spesso

Sì, ogni giorno

**In che misura stai seguendo le indicazioni ricevute dal Governo rispetto agli spostamenti?**

Molto

Abbastanza

Poco

Per niente

**Sei attualmente in isolamento domiciliare fiduciario?**

No

Sì in quanto presento sintomi suggestivi per CoVID

Sì in quanto positiva la tampone per CoVID

**Dove vivi?**

Centro di una grande città

Periferia di una grande città

Piccolo/medio centro urbano in campagna

Piccolo/medio centro urbano in località marittima

Casa isolata (o piccolo centro con poche centinaia di abitanti)

**L’appartamento in cui stai vivendo è adeguato alle tue necessità?**

Per niente

Poco

Abbastanza

Molto

Moltissimo

**Quanto è grande l’appartamento in cui vivi?**

Meno di 50 m2

Da 50 a 70 m2

Da 70 a 100 m2

Più di 100 m2

**Ci sono spazi esterni?**

No

Un piccolo balcone

Uno o più terrazzi

Giardino

**I dispositivi elettronici a cui hai accesso, e il tempo che hai per utilizzarli, sono adeguati alle tue necessità?**

Per niente

Poco

Abbastanza

Molto

Moltissimo

**I soldi che hai a disposizione, coprono tutte le tue necessità?**

Per niente

Poco

Abbastanza

Molto

Moltissimo

1.2 Aspetti psicologici per gravide e puerpere

**Quanto sei d’accordo con le seguenti affermazioni (da 1- per nulla; a 5- completamente)?**

Riesco sempre a risolvere problemi difficili se mi impegno abbastanza.

Grazie alle mie risorse, so come gestire gli imprevisti

Sono in grado di risolvere la maggior parte dei problemi se mi impegno a sufficienza.

Sono in gradi di rimanere calma quando affronto difficoltà dato che conto sulle mie capacità di adattamento

**Sei soddisfatta della tua vita?**

Estremamente

Tanto

Abbastanza

Poco

Per nulla

**Nelle ultime 2 settimane, con quale frequenza ti ha dato fastidio non riuscire a smettere di preoccuparti o a tenere sotto controllo le preoccupazioni?**

Mai

Alcuni giorni

Per più della metà del tempo

Quasi tutti i giorni

**Nelle ultime 2 settimane, con quale frequenza hai avuto i seguenti problemi? (da 0- mai; a 3- quasi tutti i giorni)**

Sentirti nervosa, ansiosa o sulle spine

Non essere in grado di controllare le tue preoccupazioni

Sentirti giù, triste o disperata

Avere scarso interesse o piacere nel fare le cose

**Quali sono le persone che senti più di sostegno in questo periodo delicato durante le restrizioni legate al decreto #iorestoacasa? *(possibile selezionare più risposte)***

Partner

Mamma

Papà

Sorelle/Fratelli

Amici

Suoceri

Altri parenti

Ostetrica/o

Ginecologa/o

Altro personale sanitario

Conoscenti in gravidanza o che hanno appena partorito

Gruppi Facebook

Siti internet

1.3 Sezione lavoro

**Qual è la tua condizione professionale?**

Disoccupata

Studentessa

Casalinga

Lavoratrice

**Di cosa ti occupi?**

Impiegata

Dirigente

Libera professionista

Operaia

Agricoltrice

Commessa

Insegnante

Professionista Sanitario

altro _____

**In questi giorni rispetto al lavoro**Lavoro da casa (smart working)

Mi reco normalmente sul posto di lavoro

Mi reco sul posto di lavoro con meno frequenza

Lavoro sia da casa sia sul luogo di lavoro

La mia attività lavorativa è sospesa

Sono stata licenziata

**Ritieni che le restrizioni attuali legate al decreto #iorestoacasa abbiano in qualche modo influito sul tuo approccio** **al lavoro?**

Sì, positivamente

Sì, negativamente

No

2.

QUESITI SOLO PER LE DONNE GRAVIDE

2.1- Anamnesi ostetrica

**È la tua prima gravidanza**

Sì

No

**A che settimana di gestazione ti trovi?**

**Indica la data presunta del parto**

**E’ una gravidanza singola o gemellare?**

- Singola

- Gemellare

**La gravidanza è insorta spontaneamente o mediante tecniche medicalmente assistite?**

Spontaneamente

Mediante tecniche medicalmente assistite

**Per quanto tempo hai cercato questa gravidanza?**

- Più di un anno
- Meno di un anno
- Meno di tre mesi
- Non è stata cercata

**Hai avuto aborti spontanei nella tua vita? (attenzione, non si includono le interruzioni volontarie di gravidanza)**

Inserire numero

**SE QUESTA NON E’ LA TUA PRIMA GRAVIDANZA**

**A quante settimane di gestazione sono nati i tuoi figli?**

**Quanto pesavano alla nascita?**

**Nelle gravidanze precedenti hai avuto complicanze? *(possibile selezionare più risposte*)**

-Diabete gestazionale

-Disturbi ipertensivi della gravidanza

-Infezioni materne in gravidanza

-Taglio cesareo urgente

-Sofferenza fetale

-Distacco di placenta

-Altro (specificare)

**Prima della gravidanza soffrivi di qualche patologia? *(possibile selezionare più risposte)***

- Diabete
- Ipertensione
- Patologie autoimmuni
- Disturbi della coagulazione
- Ansia o depressione
- Disturbi della tiroide
- Altro (specificare)

**Fumi?**

No

Sì

Ho smesso in gravidanza

**Quanto pesi? Inserisci peso attuale in kg**

**Quanto pesavi prima della gravidanza? Inserisci peso pre gravidico in kg**

**Quanto sei alta? Inserire altezza in cm**

**Fino a questo momento la tua gravidanza ha avuto qualche complicanza diagnosticata da un medico? *(possibile selezionare più risposte*)**

- Minaccia d’aborto
- Minaccia di parto prematuro
- Ipertensione gestazionale
- Diabete gestazionale
- Infezioni vaginali
- Infezione da Covid 19
- (altro specificare)

2.2- Sezione tempo libero

**Da quando è entrato in vigore il decreto #iorestoacasa quanto tempo libero hai?**

Molto meno del solito

Un po’ meno del solito

Come prima

Un po’ più del solito

Molto più del solito

**Quanto del tuo tempo libero è effettivamente a tua completa disposizione (e non utilizzato per curare i figli o dedicarsi ad altre esigenze domestiche)?**

Molto meno del solito

Un po’ meno del solito

Come prima

Un po’ più del solito

Molto più del solito

Credi che avere più tempo libero possa avere un impatto sulla gravidanza?

No

Sì, in positive

Sì, in negativo

2.3 Sezione esercizio fisico

**Indipendentemente dalle restrizioni legate al decreto #iorestoacasa ti ritieni una sportiva?**

Moltissimo

Molto

Abbastanza

Poco

Per niente

**Quanto tempo dedicavi all’esercizio fisico settimanale (incluso Yoga, camminata veloce, fitness, nuoto, etc..) prima delle restrizioni legate al decreto #iorestoacasa?**

- Più di 5 ore alla settimana
- tra 3 e 5 ore alla settimana
- tra 2 e 3 ore alla settimana
- tra mezz’ora e due ore alla settimana
- Meno di mezz’ora alla settimana

**Quanto tempo hai dedicato all’esercizio fisico nelle ultime settimane, da quando è in vigore il decreto #iorestoacasa?**

- Più di 5 ore alla settimana
- tra 3 e 5 ore alla settimana
- tra 2 e 3 ore alla settimana
- tra mezz’ora e due ore alla settimana
- Meno di mezz’ora alla settimana

**Ritieni che il fatto di essere in gravidanza abbia influito sul tuo approccio all’esercizio fisico?**

No

Sì, positivamente

Sì, negativamente

**Ritieni che le restrizioni attuali legate al decreto #iorestoacasa abbiano in qualche modo influito sul tuo approccio all’esercizio fisico?**

No

Sì, positivamente

Sì, negativamente

2.4 Sezione alimentazione

**Indipendentemente dalle restrizioni legate al decreto #iorestoacasa ti ritieni una persona che mangia sano?**

Moltissimo

Molto

Abbastanza

Poco

Per niente

**Da quando è in vigore il decreto #iorestoacasa fai più fatica ad avere una dieta sana ed equilibrata?**

Moltissimo

Molto

Abbastanza

Poco

Per niente

**Ritieni che il fatto di essere in gravidanza abbia influito sul tuo approccio all’alimentazione sana?**

No

Sì, positivamente

Sì, negativamente

**Ritieni che le restrizioni attuali legate al decreto #iorestoacasa abbiano in qualche modo influito sul tuo approccio all’alimentazione sana?**

No

Sì, positivamente

Sì, negativamente

2.5 Sezione pro e contro

**Se il tuo partner è più spesso a casa, credi che questo abbia influito sulla tua gravidanza?**

No

Sì, positivamente

Sì, negativamente

**Se hai già dei figli, credi che il fatto di averli a casa abbia influito sulla tua gravidanza?**

No

Sì, positivamente

Sì, negativamente

**Se non stai lavorando, credi che questo abbia influito sulla tua gravidanza?**

No

Sì, positivamente

Sì, negativamente

**Quanto I seguenti aspetti influenzano la tua gravidanza? (da 1- non molto a 5 – moltissimo)**

Non poter fare passeggiate

Non incontrare amici e parenti

Non poter frequentare corsi pre-parto

2.6 Sezione di accesso alle cure per gravide

**Durante le restrizioni legate al decreto #iorestoacasa hai avuto problemi che ti avrebbero indotto a recarti in un pronto soccorso ostetrico-ginecologico e hai preferito non farlo per paura del contagio?**

Si

No

**Se non ti sei recato in un pronto soccorso come hai risolto il problema?**

- contatto telefonico con ginecologo curante

- visita ginecologica privata

- visita ostetrica privata

- visita consultoriale

- consulenza telefonica con professionista ostetrico/ginecologico

- medico di medicina generale

- farmacista

- con l’aiuto di conoscenti (mamma, sorella, amica, etc)

- ho trovato la risposta su internet

- il problema si è risolto con l’attesa

- il problema non è risolto

Altro….

**Stai seguendo un corso pre-parto on-line?**

Sì

No

**Durante le restrizioni legate al decreto #iorestoacasa hai saltato alcune visite di controllo?**

Si

No

**Durante le restrizioni legate al decreto #iorestoacasa hai saltato esami o vaccinazioni programmate?**

Si

No

**Lo stato di emergenza Covid-19 ti crea stress e ansia riguardo al futuro? Quanto spesso?**

Sempre

Spesso

A volte

Raramente

Mai

**Hai delle paure legate al tuo parto?**

Moltissime

Molte

Abbastanza

Poco

Per niente

**Quali sono i tuoi principali timori legati al parto?**

_____

**Hai paura che il tuo compagno non possa essere presente quando il bambino nascerà?**

Moltissimo

Molto

Abbastanza

Poco

Per niente

**Hai paura di essere da sola con il tuo bambino quando nascerà?**

Moltissimo

Molto

Abbastanza

Poco

Per niente

**Quali sarebbero secondo te gli interventi più utili che si potrebbero implementare per rispondere alle esigenze delle gravide in questo periodo?**

**…..**

**Quali di questi interventi a tuo parere sarebbero utili anche dopo la fine dell’emergenza CoVid?**

**…..**

3. QUESITI SOLO PER LE DONNE PUERPERE

3.1 - Anamnesi ostetrica per Puerpere

**Durante la gravidanza, avevi frequentato un corso di accompagnamento alla nascita?**

Sì

No

**Avevi frequentato un corso pre-parto on-line?**

Sì

No

**Quando hai partorito?**

Data

**In quale provincia hai partorito?**

….

**E' stata la tua prima gravidanza?**

Sì

No

**Se non è stata la tua prima gravidanza, a quante settimane di gestazione sono nati gli altri tuoi figli? Per favore inserisci il numero di settimane alla nascita di ciascun figlio, in ordine cronologico, separati da uno spazio**

**……..**

**Se non è stata la tua prima gravidanza, ti ricordi quanto pesavano alla nascita gli altri tuoi figli? Per favore inserisci il peso alla nascita di ciascun figlio, in ordine cronologico, separati da uno spazio**

**……**

**Se non è stata la tua prima gravidanza, hai allattato gli altri tuoi figli? Per favore inserisci i mesi della durata dell'allattamento al seno (esclusivo o complementare) di ciascun figlio, in ordine cronologico, separati da uno spazio**

**…..**

**A quale settimana di gestazione hai partorito nell'attuale gravidanza?**

**……**

**E’ stata una gravidanza singola o gemellare?**

Singola

Gemellare

**Come è avvenuto questo parto?**

parto vaginale spontaneo eutocico

parto vaginale indotto eutocico

parto vaginale spontaneo distocico (con ventosa ostetrica)

parto vaginale indotto distocico (con ventosa ostetrica)

taglio cesareo

**Hai fatto ricorso all'analgesia del parto?**

Sì

No

**Chi era presente con te in sala parto? *(possibile selezionare più risposte*)**

Ostetrica dell’ospedale

Ostetrica privata

Ginecologo/a

Partner

Altro familiare/amico

**Prima della gravidanza soffrivi di qualche patologia? (possibile selezionare più risposte)**

Diabete

Ipertensione

Patologie autoimmuni

Disturbi della coagulazione

Ansia o depressione

Disturbi della tiroide

Altro (specificare)

**Durante questa gravidanza hai avuto qualche complicanza diagnosticata da un medico? -**

Minaccia d’aborto

Minaccia di parto prematuro

Ipertensione gestazionale

Diabete gestazionale

Infezioni

Ritardo di crescita intrauterino

Pre eclampsia

Infezione da Covid

Altro (specificare)

**Quanti giorni è durato il ricovero in ospedale dopo il parto?**

……..

**Hai avuto qualche problema/complicanza dopo il parto?**

……….

**Il tuo bambino ha necessitato di cure neonatologiche intensive?**

Sì

No

**E' stato ricoverato in Patologia Neonatale/Terapia Intensiva Neonatale?**

Sì

No

**Se sì, per quale problema?**

**……**

**Se sì, quanto è durata la sua degenza?**

(numero di giorni)…..

3.2 Sezione allattamento

**Dopo il parto, hai iniziato ad allattare al seno il tuo bambino?**

sì, già in sala parto, nelle prime 2 ore dopo la nascita

sì, in prima giornata di vita

sì, nei giorni successivi

no, ho preferito non farlo

no, ho riscontrato difficoltà

**Durante il ricovero in ospedale, il tuo bimbo ha mangiato?**

solo il mio latte al seno

solo il mio latte al seno o spremuto

anche latte formulato

anche latte umano donato

solo latte formulato

altro (specificare)

**Chi ti ha seguito per l'allattamento durante la degenza? *(possibile selezionare più risposte)***

Ostetrica

Puericultrice

Infermiere

Ginecologo

Neonatologo

Nessuno

**Al momento della dimissione, ti sei sentita autonoma rispetto all'allattamento?**

Sì

No

**Dopo la dimissione, hai allattato il tuo bambino?**

Sì

No

**Stai ancora allattando il tuo bambino?**

Sì

No

**Dopo la dimissione, sei stata aiutata da qualcuno per allattare il tuo bambino? *(possibile selezionare più risposte)***

Nessuno

Partner

Madre

Suocera

Altro familiare/amica

Ostetrica del consultorio

Ostetrica del punto nascita

Ostetrica privata

Ginecologo

Pediatra

Altro (specificare)

**Ritieni che le restrizioni attuali legate al decreto #iorestoacasa abbiano in qualche modo influito sul tuo allattamento?**

No

Sì, positivamente

Sì, negativamente

**Perché?**

(Specificare)

3.3 Sezione gestione del neonato

**Durante il ricovero, il tuo bimbo è stato visitato dal pediatra/neonatologo?**

Sì, quotidianamente

Sì, solo alla nascita

Sì, solo alla dimissione

No

Non so

**In caso tu fossi positiva al CoVid, sei stata separata dal tuo bambino dopo la nascita?**

Sì

No

**In caso tu fossi positiva al CoVid, hai avuto la possibilità di dare il tuo latte al tuo bambino dopo la nascita?**

Sì

No

**Durante la degenza, sei diventata autonoma nel cambio/igiene del tuo bambino?**

Sì

No

**Durante il ricovero, sei diventata autonoma nella medicazione del moncone ombelicale del tuo bambino?**

Sì

No

**Dopo la dimissione, sei stata aiutata da qualcuno nella cura/cambio/igiene del tuo bambino? *(possibile selezionare più risposte)***

Partner

Madre

Suocera

Altro familiare/amica

Altro (specificare)

Nessuno

**Nella settimana successiva alla dimissione, il tuo bambino è stato sottoposto a visite mediche? *(possibile selezionare più risposte)***

No

Sì, presso il punto nascita

Sì, presso il consultorio

Sì, presso il pediatra curante

Sì, presso il pronto soccorso pediatrico

**Ritieni che le restrizioni attuali legate al decreto #iorestoacasa abbiano in qualche modo influito sulla gestione del tuo bambino?**

No

Sì, positivamente

Sì, negativamente

3.4 Aspetti psicologici per puerpere

**Eri preoccupata all'idea di partorire durante la pandemia?**

Molto

Abbastanza

Poco

Per nulla

**Temevi che avresti ricevuto minore assistenza durante il ricovero?**

Sì

No

**Rispetto alle tue aspettative circa l’assistenza a te, la realtà è stata:**

Migliore

Come immaginavo

Peggiore

**Temevi che il tuo bambino avrebbe ricevuto minore assistenza durante il ricovero?**

Sì

No

**Rispetto alle tue aspettative circa l’assistenza al tuo bambino, la realtà è stata:**

Migliore

Come immaginavo

Peggiore

**Le indicazioni restrittive del decreto #iorestoacasa rispetto agli spostamenti, hanno avuto impatto sul tuo umore una volta dimessa dall'ospedale?**

Sì

No

**In che modo?**

**……..**

**Se non fosse stato in vigore il decreto #iorestoacasa, pensi che a casa dopo il parto:**

avresti ricevuto più aiuti

avresti ricevuto meno aiuti

non sarebbe cambiato nulla

**Al ritorno a casa dall'ospedale, ti sei sentita capace/adeguata rispetto alla gestione del tuo bambino?**

Molto

Abbastanza

Poco

Per niente
